# Bioactive Hydrogel Scaffolds Integrating Chitosan, Silk Fibroin, and *Aloe vera* Extract for Enhanced Cartilage Tissue Regeneration

**DOI:** 10.3390/polym17101409

**Published:** 2025-05-20

**Authors:** Witwisitpong Maneechan, Phassorn Khumfu, Pensri Charoensit, Areeya Tuanchai, Sukunya Ross, Gareth M. Ross, Jatuporn Ngoenkam, Jarupa Viyoch

**Affiliations:** 1 Department of Pharmaceutical Technology, Faculty of Pharmaceutical Sciences, Naresuan University, Phitsanulok 65000, Thailand; witwisitpong.ma@nu.ac.th (W.M.); phassornk66@nu.ac.th (P.K.); pensric@nu.ac.th (P.C.); 2 Center of Excellence in Biomaterials, Department of Chemistry, Faculty of Science, Naresuan University, Phitsanulok 65000, Thailand; areeya.tu@nu.ac.th (A.T.); sukunyaj@nu.ac.th (S.R.); gareth@nu.ac.th (G.M.R.); 3 Department of Microbiology and Parasitology, Faculty of Medical Science, Naresuan University, Phitsanulok 65000, Thailand

**Keywords:** hydrogel scaffold, chitosan, silk fibroin, aloe vera, cartilage tissue

## Abstract

This study developed composite hydrogel scaffolds from chitosan (CS), silk fibroin (SF), and *Aloe vera* (AV) gel extract for cartilage tissue engineering. SF extracted from Nang-Laai silkworm cocoons showed high protein content (86.8%), while AV extract contained characteristic polysaccharides. Scaffolds with varying CS/SF/AV ratios were fabricated and evaluated for physicochemical and biological properties. Among all formulations, CS40/SF/AV (3.00%wt CS, 2.70%wt SF, 0.075%wt AV) exhibited superior porosity (72.23 ± 4.85%), pore size (79.57 ± 3.68 μm), and compressive strength, both in dry (6.67 ± 1.44 MPa) and wet states. It also showed controlled swelling (270%) and a stable degradation profile (55–57% over 21 days). FTIR and XRD confirmed successful component integration and semi-crystalline structure. In vitro, CS40/SF/AV supported chondrocyte adhesion, proliferation, and morphology retention over 28 days. Fluorescence imaging showed uniform cell distribution across the scaffold. These results highlight the CS40/SF/AV scaffold as a promising, biocompatible platform with optimal mechanical and structural properties for cartilage regeneration, offering potential for further in vivo applications.

## 1. Introduction

Osteoarthritis is a major cause of mobility limitations, especially degeneration in the knee joint. Data indicate that between 2007 and 2017, knee osteoarthritis increased by 30.8%, which represents a significant increase for a non-communicable disease [1]. The number of patients with this disease is expected to increase significantly in the coming years due to an aging population and increasing prevalence of overweight conditions. In Thailand, reports show that in 2018, the incidence of knee osteoarthritis was approximately 8.64%, and healthcare expenditures for treating this disease are relatively high [2]. Treatment options for osteoarthritis include both conservative and invasive approaches. Conservative treatment focuses on symptomatic management, primarily using non-steroidal anti-inflammatory drugs (NSAIDs) to reduce pain and inflammation in affected joints. Beyond medication, treatment may involve artificial synovial fluid injections to improve joint lubrication and various surgical procedures designed to repair damaged cartilage in the affected joints. However, the outcomes from these treatment methods or techniques are often unpredictable, and in many cases, they do not achieve long-term clinical success. The degeneration and wear of articular cartilage occur due to several factors. First, the thickness of the cartilage layer covering the joint surface decreases. Second, the cartilage loses its essential physical and chemical properties. These changes primarily result from a reduction in the number of cartilage cells, known as chondrocytes. Additionally, these cells produce less extracellular matrix, particularly important components like collagen and the proteoglycans, which provide strength and flexibility to the cartilage structure. Consequently, tissue engineering techniques have been conceptualized to repair degenerated or worn joint cartilage.

The factors that promote success in cartilage tissue engineering development are as follows: (1) scaffolds that provide a structure for chondrocyte attachment and function, (2) sources of chondrocytes or stem cells, (3) essential substances that promote chondrocyte growth and function. Scaffolds are often prepared from polymers that are biocompatible and biodegradable in the human body. Additionally, they should be capable of being prepared in forms with physicochemical properties suitable for cartilage tissue engineering. For example, hydrogel scaffolds need appropriate strength, flexibility, and biodegradation rates. These polymers may be obtained from synthesis or natural sources. Examples of polymers used in scaffold preparation include poly (glycolic acid), poly (lactic acid), and poly (lactic-co-glycolic acid) [3,4,5]. These synthetic polymers are relatively costly, which contributes to the high expenses associated with cartilage tissue engineering procedures. In Thailand, the service fee for artificial knee replacement using scaffolds that may or may not contain cells, such as chondrocytes or stem cells, costs approximately USD 5714 per side. Based on these considerations, this research explores the use of natural polymers derived from raw materials and produced domestically. These polymers are combined with essential substances which are natural extracts from Thai herbs. These herbs help promote the growth and function of chondrocytes.

In this study, chitosan derived from marine animal shells, such as shrimp shells, and fibroin extracted from *Bombyx mori* cocoons were utilized as scaffold materials. Both natural polymers are known for their biocompatibility and biodegradability in physiological conditions. When combined at optimal ratios and cross-linked between polymer chains, chitosan and fibroin form hydrogels exhibiting outstanding mechanical strength and flexibility. Additionally, the fluid-retaining properties of these hydrogels facilitate nutrient delivery from cell culture media or physiological fluids to embedded living cells. Previous research has demonstrated that fibroin promotes cellular growth [6,7,8]. Fibroin is composed of alternating hydrophobic and hydrophilic segments. The hydrophobic blocks are rich in amino acids with small side chains, such as glycine and alanine, which promote the formation of β-sheet structures through intermolecular hydrogen bonding. These highly ordered β-sheets are responsible for the remarkable mechanical strength of silk fibroin. In contrast, the hydrophilic regions contain amino acids with larger or charged side chains, resulting in a more disordered, amorphous structure. This combination of crystalline and amorphous domains imparts both strength and flexibility to fibroin, making it well suited for the development of biomedical materials. However, one problem with fibroin when made into a hydrogel is that the molecular chains of fibroin tend to aggregate into large β-sheet structures. This aggregation occurs through conformational changes from random coil to β-sheet structure, and such changes affect the mechanical properties of the hydrogel during use. The solution is to mix fibroin with other polymers, such as chitosan [9,10], in appropriate proportions. This approach should be combined with using cross-linking agents [10,11]. These strategies help ensure that the good properties of each polymer can be maintained. Chitosan is a promising biopolymer derivative of chitin, a polysaccharide found abundantly in marine animal shells. Through chemical procedures that remove the acetyl group from chitin, chitosan is obtained with remarkable structural and functional properties. Its hydrophilic nature stems from the presence of a hydroxyl group at the 6-carbon position and an amino group at the 2-carbon position, which determine its specific applications in tissue engineering. Chitosan exhibits several beneficial characteristics: it is nontoxic, sensitive to pH, renewable, biocompatible, biodegradable, bi-functional, and non-antigenic [12]. When dissolved in acidic water, chitosan carries a positive charge and transforms into a gel-like state as the environment becomes more alkaline (pH greater than 6). This pH-responsiveness is particularly valuable for cartilage tissue engineering applications. The positively charged portions of chitosan can effectively bind to the negative charges of glycosaminoglycans (GAGs), which are components of the proteoglycan framework in cartilage. This electrostatic interaction stimulates chondrocyte function and promotes cartilage tissue formation [13,14]. Due to these advantageous properties, chitosan has been widely incorporated in polymer composite systems and combined with inorganic particles, making it an excellent candidate for scaffolds in cartilage tissue regeneration.

In addition to scaffolds, a crucial component for creating cartilage tissue is essential substances that promote growth and/or reduce the death of chondrocytes in abnormal conditions. Previous studies found that hydrogels prepared from silk fibroin combined with *Aloe vera* gel extract can heal wounds in mice induced with diabetes. This healing effect is a result of fibroblast cells being stimulated by substances in *Aloe vera* gel. The clear gel inside *Aloe vera* leaves contains small molecular weight polypeptide proteins (20–100 kDa) with anti-inflammatory properties [8].

The structure of these polypeptides includes serine carboxypeptidase, an enzyme that can cleave the C-terminal of bradykinin molecules and reduce the secretion of the inflammatory mediator TNF-α [15,16]. It also contains glucomannans, which are complex carbohydrates or polysaccharides. These compounds help inhibit the activity of cyclooxygenase enzymes [16]. This inhibition blocks the synthesis of prostaglandins, which are primary mediators in the inflammatory process. Therefore, the objective of this research is to develop hydrogel scaffold materials from chitosan and fibroin, containing *Aloe vera* gel extract, for use in cartilage tissue replacement. The efficacy of these scaffold materials for cartilage tissue replacement will be evaluated based on their ability to promote cartilage tissue formation at the cellular level.

## 2. Materials and Methods

### 2.1. Materials and Reagents

Shrimp shell-derived chitosan (MW 10^5^–10^6^ Da, deacetylation degree > 90%) was obtained from Sinudom Agriculture Products Ltd. (Suratthani, Thailand). The Center for Excellence in Silk (Nakhon Phathom Province, Thailand) provided *Bombyx Mori* (Nang Laai strain) yellow silkworm cocoons. Fresh *Aloe vera* plants were harvested from cultivation sites in Phitsanulok Province, Thailand. For chemical reagents, the following were utilized: Sigma-Aldrich Chemie GmbH (Steinheim, Germany) supplied sodium bicarbonate, phosphate-buffered saline, and 88% lactic acid solution. Di-sodium hydrogen phosphate (Na_2_HPO_4_) was acquired from Elago Enterprises Pty Ltd. RCI Labscan (Bangkok, Thailand) provided calcium chloride (CaCl_2_), sodium hydroxide (NaOH), and ammonium sulfate ((NH_4_)_2_SO_4_). Dialysis membrane standard RC tubing (MWCO: 6–8 kDa) was sourced from Spectrum Laboratories, Inc. (Rancho Dominguez, CA, USA). Additional materials included Pierce™ BCA Protein Assay Kit (Thermo Fisher Scientific Inc., Rockford, IL, USA); Modified Eagle’s Medium (DMEM), fetal bovine serum (FBS), and 0.25% trypsin/0.01M EDTA (Sigma-Aldrich Co., St. Louis, MO, USA); Gibco™ Penicillin–Streptomycin (10,000 U/mL) (Thermo Fisher Scientific Inc.); cell viability reagent (Thiazolyl Blue Tetrazolium Bromide, MTT) and paraformaldehyde (Sigma-Aldrich); lysozyme (HiMedia Laboratories Pvt. Ltd., Lincoln University, PA, USA); and ProLong™ Diamond Antifade Mountant with DAPI (Thermo Fisher Scientific Inc.).

### 2.2. Preparation and Characteristic Determination of the Silk Fibroin and Aloe Vera Extract

Silk fibroin and *Aloe vera* gel extracts were prepared following a modified methodology described in a previous study [8]. Briefly, silk fibroin was extracted from yellow silkworm cocoons (*Bombyx Mori*, Nang-Lai strain) through a multi-step process. Initially, the raw cocoons were boiled in hot deionized water for 2 h. Then, the material was further boiled in NaOH solution for 30 min. The degummed fiber was rinsed with DI water 3 times and dried overnight. The dried fibers were dissolved in CaCl_2_ solution at 90 °C for 4–6 h, followed by dialysis. Finally, the solution underwent lyophilization (freeze-drying) and the resulting silk fibroin was stored in a desiccator.

Meanwhile, the extraction of *Aloe vera* gel involved several sequential steps. Briefly, fresh *Aloe vera* leaves were initially peeled to obtain the gel portion, which was then blended into a homogeneous mixture. Salt ((NH_4_)_2_SO_4_) was added to the mixture for precipitation of proteins and other compounds. The resulting crude extract was collected and dissolved in deionized water with constant stirring. This solution was subjected to dialysis to desalt the extract. Finally, the purified solution underwent lyophilization and the product was stored in a desiccator for future use.

For both silk fibroin and *Aloe vera* gel extracts, characterization involved several analytical techniques. Pierce™ BCA Protein Assay Kit (Thermo Fisher Scientific Inc., Waltham, MA, USA) was employed to quantify protein content, while molecular weight distribution was evaluated through sodium dodecyl sulfate polyacrylamide gel electrophoresis (SDS-PAGE). Chemical composition and structural features were identified using Fourier transform infrared spectroscopy (FTIR spectrometer, Spectrum GX series, PerkinElmer Inc., Waltham, MA, USA).

### 2.3. Fabrication of CS/SF/AV Hydrogel Scaffolds

The composite scaffolds of chitosan/silk fibroin/*Aloe vera* (CS/SF/AV) were prepared with different concentrations (Table 1). Briefly, CS solutions in 0.5 M acetic acid were adjusted to pH 5–6 using 10% NaHCO_3_ (*w*/*v*) solution, then blended with SF solutions in varying ratios (20:55.46, 30:45.73, and 40:36) with lyophilized *Aloe vera* to obtain final concentrations of CS (1.50–3.00% *w*/*v*), SF (2.70–4.16% *w*/*v*), and AV (0.075–0.12% *w*/*v*). All scaffolds maintained a constant cross-link concentration of 2.25% *w*/*v*. This CS/SF/AV composite mixture was poured into a 24 multiwell plate and left overnight in a −80 °C freezer, prior to drying by lyophilization. After lyophilization, the samples were stored in a desiccator for experimental use. Similarly, the control scaffold containing only chitosan (5% *w*/*v*) without natural extracts was also lyophilized in the same manner.

All materials and composite scaffolds were characterized using FTIR spectrometer and X-ray diffraction (XRD). The morphologies of composite scaffolds were studied using scanning electron microscope (Model 1455VP, LEO Electron Microscopy Ltd., Cambridge, UK).

Pore size and morphological characteristics of the scaffolds were evaluated using scanning electron microscopy (SEM). Images obtained from SEM were analyzed with ImageJ software (version 1.54g) to determine the average pore diameter and porosity. For each scaffold formulation, measurements were taken from at least 50 pores in triplicate samples.

The water absorption capacity of the scaffolds was evaluated based on methods reported by Felfel et al. [17]. In summary, the initial mass of dried scaffolds was measured (W_0_) before immersion in phosphate-buffer solution (PBS) at pH 7.4 maintained at 37 °C for a 24 h period. After incubation, triplicate scaffold samples (*n* = 3) were extracted from the PBS medium, carefully dabbed with filter paper to eliminate surface moisture, and their hydrated mass (W1) was documented. The degree of swelling was then calculated using Equation (1).

Swelling ratio = ((W_1_ − W_0_)/W_0_) × 100 (1)

Scaffold mechanical behavior was characterized through compression testing under non-confined conditions using a TA.XT plus Texture Analyser system (Microsystems Ltd., London, UK) with compression rate set at 1 mm/min and a 30 kg capacity load cell. Sample dimensions were determined by averaging three diameter and height measurements for each cylindrical specimen, with these dimensional parameters and cross-sectional area incorporated into the testing software (Texture Exponent version 6.1.20.0) prior to analysis. During compression, the system continuously monitored and recorded force (N) and positional displacement (mm) data. From these measurements, engineering stress (σ) and strain (ε) values were derived and plotted as stress–strain relationships. Specimens underwent compression to 60% of their original height. Elastic modulus was determined from the initial linear region of the stress–strain curve (up to 2% strain), while maximum compressive strength was identified at 20% deformation for consistency with previous reports [18]. To assess performance under physiologically relevant conditions, identical testing was conducted on samples after 24 h PBS immersion to ensure complete hydration. All formulations were evaluated in triplicate under both dry and hydrated states (*n* = 3).

The enzymatic degradation behavior of composite scaffolds was evaluated in a physiological environment using PBS supplemented with lysozyme (10,000 U/mL) at physiological temperature (37 °C), adapting methodology from Farshi Azhar et al. [19]. Test specimens were submerged in the enzyme solution and monitored over periods of 7, 14, and 21 days. Prior to immersion, samples were weighed to establish initial mass (W_0_). Following each time point, samples were rinsed thoroughly with distilled water to eliminate adsorbed ions from the surface and subsequently freeze-dried. The resulting mass was recorded as W_t_. The percentage of scaffold degradation was determined according to Equation (2).

Degradation (%) = [(W_0_ − W_t_)/W_0_] × 100 (2)

### 2.4. Chondrocytes Culture and Seeding

Human primary chondrocyte cell line (HUM-iCell-s018, iCell, China) was acquired from iCell Bioscience Inc., Shanghai, China. Cells were maintained in the Primary Chondrocyte Culture System (PriMed-iCell-020, iCell) at 37 °C under 5% CO_2_ atmospheric conditions. Culture medium was refreshed every 3 days. Upon reaching 70–80% confluence, cells were harvested using Trypsin-EDTA solution (Sigma-Aldrich Co., St. Louis, MO, USA) for subculture. For experimental procedures, cells at passage seven were seeded onto the scaffold constructs in culture plates and maintained under standard incubation conditions.

Cellular proliferation on scaffold constructs was evaluated using the MTT colorimetric assay. Prior to cell seeding, scaffold materials underwent sterilization in 70% ethanol solution for 15 min followed by 3 h of sterile PBS rinsing. Scaffold conditioning was performed through 12 h pre-incubation in complete culture medium to ensure saturation. Subsequently, scaffolds were transferred to culture plates with appropriate media. A suspension containing 3 × 10^4^ cells was applied drop-wise onto each scaffold’s upper surface, allowing cellular distribution throughout the porous structure. To promote cell attachment, seeded scaffolds were maintained at 37 °C in a humidified 5% CO_2_ atmosphere for 6 h. Culture medium was refreshed every 48 h, with experimental assessments conducted on days 1 and 7 post-seeding.

At designated timepoints, culture media were aspirated from the scaffolds and replaced with fresh medium containing MTT solution (0.5 mg/mL). Samples were then incubated for 4 h at 37 °C in a 5% CO_2_ humidified environment. During this period, viable cells converted MTT through mitochondrial enzyme activity, resulting in the formation of purple formazan precipitates. Following incubation, the medium with formazan crystals was extracted, and the precipitated formazan was solubilized in DMSO. After complete dissolution, absorbance measurements of the supernatant were taken at 540 nm using a microplate reader (Spectra Count, Perkin Elmer, Waltham, MA, USA). Scaffold constructs without cells were processed identically and used as background controls. All measurements were conducted in triplicate, with results expressed as absorbance values. Data from 3D scaffold cultures were analyzed in comparison to conventional monolayer (2D) culture conditions.

Cellular morphology on scaffold surfaces was examined using scanning electron microscopy (SEM). The representative scaffold formulation with optimal properties was collected after 7 days of cell culture and fixed using 4% *w*/*v* paraformaldehyde solution (Sigma-Aldrich). Samples were subsequently lyophilized, coated with a thin gold layer via sputter-coating technique, and then visualized under SEM.

Cell nuclei were stained using ProLong™ Diamond Antifade Mountant with DAPI (Thermo Fisher Scientific, Waltham, MA, USA). Briefly, the scaffold materials with cells cultured for 28 days were smeared onto glass slides. Cells were fixed using 4% paraformaldehyde (pH 7.4) for 10 min at 37 °C. This was followed by permeabilization with 0.1% Triton X-100 in 1X PBS (Sigma-Aldrich Co., St. Louis, MO, USA) and incubation at room temperature for 15 min. Subsequently, one drop of ProLong™ Diamond Antifade Mountant with DAPI was added onto the glass slide. Following a 24 h curing period in darkness at ambient temperature, the prepared slides were visualized and documented using an Axio Observer Z1 inverted fluorescence microscope (Carl Zeiss AG, Oberkochen, Germany).

### 2.5. Statistical Analysis

Data are presented as mean ± standard deviation (SD). For comparing two independent groups, Student’s *t*-test was applied. Multiple group comparisons were conducted using analysis of variance (ANOVA), with the Kruskal–Wallis H test employed for datasets that did not meet normality assumptions (as determined by Shapiro–Wilk’s test). Statistical significance was established at *p* < 0.05. Statistical computations were performed using Statistical Package for Social Science (SPSS) software version 19 for Windows.

## 3. Results and Discussion

### 3.1. The Characteristics of the Silk Fibroin and Aloe Vera Gel Extract

**Figure 1:**
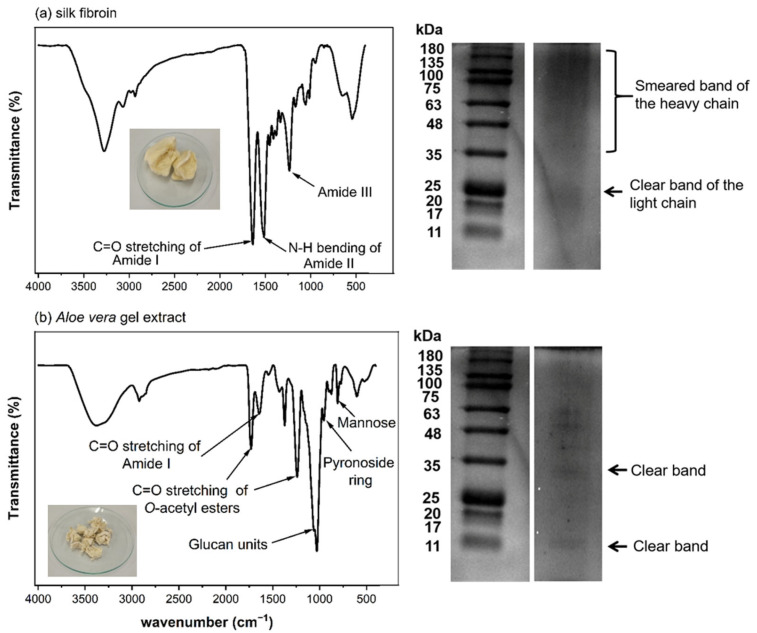
Characterization of silk fibroin and *Aloe vera* gel extract. (**a**) Silk fibroin analysis showing FTIR spectrum with characteristic amide peaks, protein profile by SDS-PAGE, and physical appearance. (**b**) *Aloe vera* gel extract characterization displaying FTIR spectrum with key functional groups, protein bands by SDS-PAGE, and freeze-dried extract appearance.

Freeze-dried silk fibroin extract obtained from Nang-Laai strain silkworm cocoons exhibited a yellowish, cotton-like appearance. The extraction process yielded 0.43 g of material per gram of cocoons, representing a 43% *w*/*w* recovery rate. Analysis of protein content via protein assay revealed that 86.8 ± 2.6% *w*/*w* of the extract consisted of protein. FTIR spectroscopic analysis identified characteristic frequency peaks at 1631 (amide I), 1513 (amide II), and 1232 (amide III) cm^−1^. Molecular weight distribution analysis demonstrated a distinct L-chain band at approximately 25 kDa, with H-chain components appearing as a smear band ranging from 30 to 245 kDa (Figure 1a).

Freeze-dried Aloe vera gel extract appeared as a white cotton-like substance, with an extraction efficiency of 1.03% w/w relative to the starting material. Protein analysis determined that protein components constituted 4.9–7.2% *w*/*w* of the total extract. Spectroscopic characterization via FTIR identified signature absorption bands at 1731 and 1239 cm^−1^ (corresponding to O-acetyl ester groups), 1059 cm^−1^ (associated with glucan units), 955 cm^−1^ (indicative of pyranoside ring structures), and 807 cm^−1^ (attributed to mannose). Electrophoretic analysis revealed distinct protein bands at approximately 14 and 35 kDa in the molecular weight profile (Figure 1b).

The characterization results show that both the prepared silk fibroin and *Aloe vera* gel extract have chemical compositions consistent with previous reports [8,16,20]. They contain important components that may contribute to the biological properties of the developed scaffold materials. In particular, the compounds in *Aloe vera* with anti-inflammatory and wound-healing properties could be beneficial for cartilage tissue formation and repair.

### 3.2. Morphology and Characteristics of Composite Scaffolds

**Figure 2:**
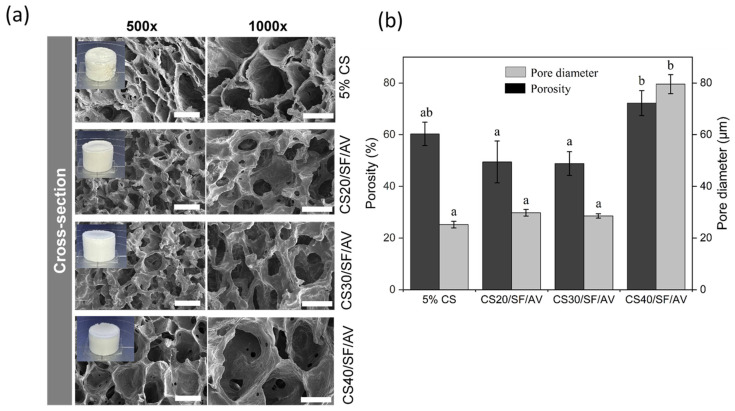
The morphology and characteristics of composite scaffolds: (**a**) An SEM image of the cross-section of the scaffolds at magnifications of 500× (scale bar = 50 μm) and 1000× (scale bar = 30 μm) and (**b**) the average of the porosity measurements and pore diameter of the scaffolds. The error bars indicate standard deviation. Statistical significance (*p* < 0.05) between groups is denoted by different letters above bars, as determined through Dunn’s multiple comparisons post hoc analysis.

The cross-sectional SEM images of CS/SF/AV scaffolds are shown in Figure 2a, illustrating the influence of varying CS/SF/AV composition ratios on pore architecture and septum thickness. The scaffolds exhibited distinct morphological characteristics: CS20/SF/AV and CS30/SF/AV showed similar porosity (49.44 ± 8.10% and 48.82 ± 4.60%, respectively) and pore diameters (29.82 ± 1.26 μm and 28.57 ± 0.82 μm, respectively). In contrast, the CS40/SF/AV scaffold demonstrated significantly enhanced porosity (72.23 ± 4.85%) and a larger average pore diameter (79.57 ± 3.68 μm). The control scaffold exhibited moderate porosity (60.27 ± 4.53%) with the smallest pore diameter (25.20 ± 1.26 μm). The Kruskal–Wallis H test revealed significant differences in the mean porosity and pore diameter among formulations (chi-squared = 8.897, *df* = 3, *p* = 0.031; chi-squared = 9.667, *df* = 3, *p* = 0.022, respectively). Subsequent Dunn’s post hoc analysis indicated that CS40/SF/AV exhibited significantly higher porosity compared to CS30/SF/AV and CS20/SF/AV formulations (*p* < 0.05), while no significant differences were observed between the control group (5% CS) and other formulations. For pore diameter, CS40/SF/AV demonstrated significantly larger pores compared to the control group (*p* < 0.05), with no significant differences noted among the other formulations (Figure 2b).

These results indicate that increasing the chitosan content within the CS/SF/AV blend promotes the formation of more porous structures with larger pores. This trend aligns with the findings of Qi et al. [21], who reported that higher chitosan ratios in chitosan/silk fibroin scaffolds resulted in porosity values ranging from 90.5% to 96.1%, with pore sizes between 100 and 300 μm, depending on the material composition and the size of the porogen used. Although the absolute values differ, both studies support the conclusion that the CS/SF ratio is a key determinant in scaffold morphology, which plays a crucial role in facilitating cell infiltration and tissue regeneration.

**Figure 3:**
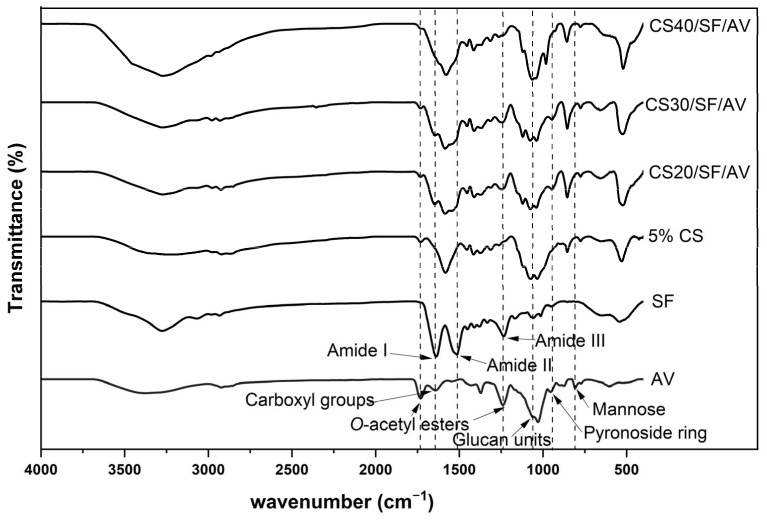
FTIR spectra of pure components (SF and AV) and composite scaffolds.

The FTIR spectra of various chitosan-based composites were examined to identify their characteristic functional groups. The spectra presented in Figure 3 show the transmittance (%) versus wavenumber (cm^−1^) in the range of 4000–500 cm^−1^ for six different samples: CS40/SF/AV, CS30/SF/AV, CS20/SF/AV, 5% CS, SF, and AV. The spectra of CS40/SF/AV, CS30/SF/AV, and CS20/SF/AV composites exhibited similar characteristic peaks with varying intensities corresponding to their chitosan content. All chitosan-containing samples displayed characteristic absorption bands of amide groups, with Amide I, II, and III bands visible in the 1700–1200 cm^−1^ region. The carbonyl groups were observed at approximately 1700 cm^−1^, while carboxyl group of O-acetyl esters were identified around 1650–1600 cm^−1^. The characteristic peaks for glucan units were detected in the 1500–1400 cm^−1^ region. Additionally, mannose and pyranoside ring structures were observed at 900–800 cm^−1^ and 800–700 cm^−1^, respectively, confirming the polysaccharide nature of the chitosan component. The pure SF (silk fibroin) sample displayed its distinctive peaks, particularly in the amide regions, while the AV (*Aloe vera*) sample showed characteristic absorption bands related to its polysaccharide composition. The 5% CS sample exhibited characteristic chitosan peaks but with lower intensity compared to the higher concentration samples.

**Figure 4:**
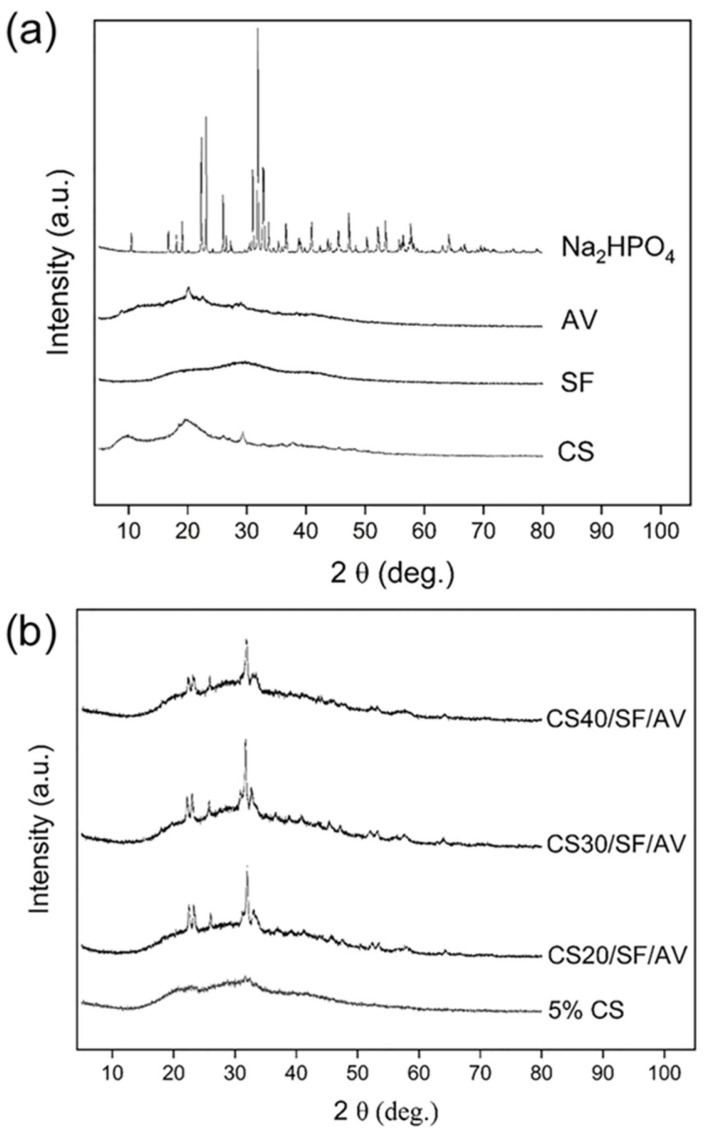
XRD patterns of (**a**) chitosan, silk fibroin, *Aloe vera* gel extract, disodium hydrogen phosphate, and (**b**) 5% chitosan (control), CS20/SF/AV, CS30/SF/AV, and CS40/SF/AV scaffolds.

Figure 4a shows the XRD patterns of individual raw materials. Chitosan exhibited broad crystalline peaks with low intensity at approximately 10°, 20°, and 29°, indicating the presence of both crystalline and amorphous regions. Silk fibroin displayed broader and lower-intensity peaks, characteristic of an amorphous structure. Similarly, *Aloe vera* gel extract exhibited broad peaks with low intensity, along with a sharper crystalline peak around 20°, suggesting a semi-crystalline nature. In contrast, disodium hydrogen phosphate displayed several sharp characteristic peaks, particularly at 2θ angles around 20° and 30°, indicating its crystalline structure. In Figure 4b, the XRD patterns of the fabricated scaffolds are presented. Starting with 5% CS, which showed broad crystalline peaks characteristic of both crystalline and amorphous structure, the XRD patterns of scaffolds with various ratios of CS (CS20/SF/AV, CS30/SF/AV, and CS40/SF/AV) were analyzed. The composite scaffolds maintained a semi-crystalline nature, exhibiting broad peaks with low intensity. However, slightly more pronounced peaks were observed at 2θ angles between 20 and 25°. Notably, the peak intensity showed an increasing trend with higher chitosan content in the composite scaffolds.

FTIR and XRD analyses confirmed the successful integration of all components within the composite scaffolds. The maintenance of semi-crystalline nature across different formulations, as evidenced by broad peaks with low intensity in XRD patterns, represents a critical advantage for tissue engineering applications. This semi-crystalline structure provides an optimal balance between mechanical stability and flexibility, unlike highly crystalline materials which tend to be rigid but brittle [22,23]. The semi-crystalline structure of the scaffolds—resulting from the combined use of CS, SF, and AV—is anticipated to promote controlled biodegradation, allowing for gradual replacement by native tissue while maintaining structural integrity during the regeneration process [24,25]. Additionally, this structural characteristic enhances cellular adhesion and interaction by providing a biomimetic environment that more closely resembles the partially ordered structure of natural extracellular matrix in cartilage tissue [26,27].

**Figure 5:**
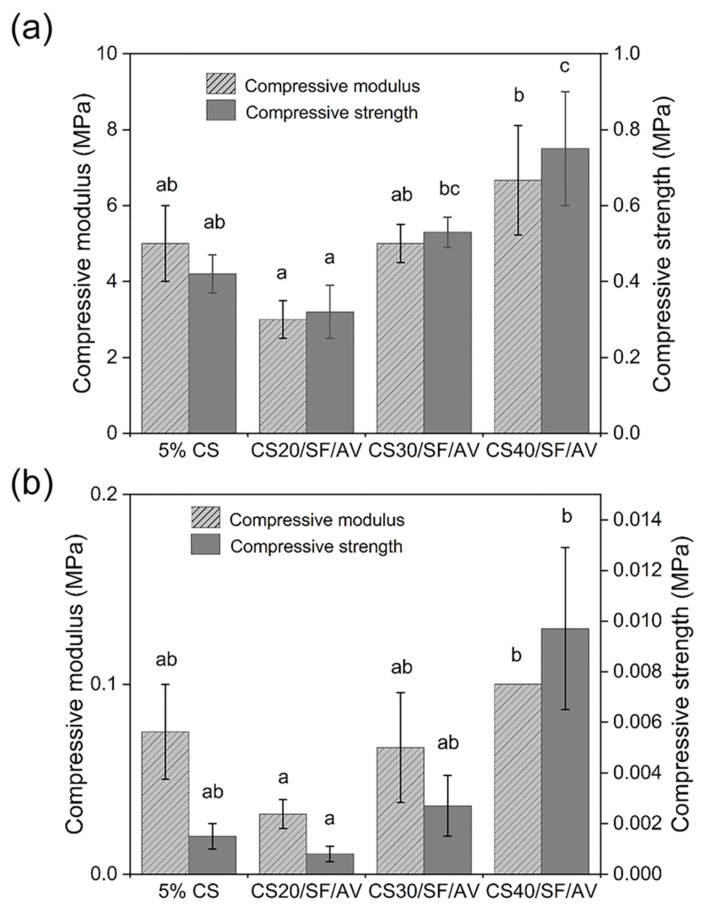
Mechanical compression analysis of scaffold formulations under (**a**) dry conditions and (**b**) hydrated state following 24 h PBS immersion. For each composition, three replicates (*n* = 3) were evaluated, with compressive modulus and strength calculated using standardized testing protocols. Error bars indicate standard deviation. Statistical significance (*p* < 0.05) between groups is denoted by different letters above bars, as determined through Dunn’s multiple comparisons post hoc analysis.

The mechanical properties of the scaffolds were investigated through unconfined compression tests in dry conditions, analyzing stress–strain curves within 2% strain for compressive modulus calculation and at 20% strain for stress measurement. The compressive properties of scaffolds were evaluated under both dry and wet conditions, as shown in Figure 5. Under dry conditions (Figure 5a), the CS40/SF/AV composition demonstrated superior mechanical properties with the highest compressive modulus (6.67 ± 1.44 MPa) and compressive stress (0.75 ± 0.15 MPa), while CS20/SF/AV exhibited the lowest values (3.00 ± 0.50 MPa modulus). A general trend of increasing mechanical properties was observed with higher CS content. Kruskal–Wallis H test revealed significant differences in the mean of compressive modulus and compressive stress among formulation under dry conditions (chi-squared = 7.751, *df* = 3, *p* = 0.05; chi-squared = 10.019, *df* = 3, *p* = 0.01, respectively).

When tested under wet conditions (Figure 5b), all scaffold compositions showed significantly reduced mechanical properties, with values approximately an order of magnitude lower than their dry counterparts. However, CS40/SF/AV maintained its superior performance relative to other compositions even in wet conditions, showing the highest compressive modulus (0.10 MPa) and stress (0.01 MPa). Kruskal–Wallis H test revealed significant differences in the mean of compressive modulus and compressive stress among formulation under wet conditions (chi-squared = 8.369, *df* = 3, *p* = 0.03; chi-squared = 9.787, *df* = 3, *p* = 0.02, respectively).

The mechanical properties assessment revealed a clear correlation between chitosan content and mechanical strength. This enhancement is attributed to chitosan’s rigid polysaccharide backbone and its ability to form extensive hydrogen bonding networks, which contribute to the structural integrity of the matrix. This finding is supported by Silva et al. [28] who reported that in chitosan–siloxane hybrids, increasing chitosan concentration significantly enhances the material’s mechanical properties. In particular, the compression modulus increases proportionally with the chitosan content. Under dry conditions, CS40/SF/AV demonstrated superior compressive modulus (6.67 ± 1.44 MPa) and compressive stress (0.75 ± 0.15 MPa), which aligns with findings by Mirahmadi et al. [29], who reported that reinforcing chitosan hydrogels with silk fibers significantly enhances mechanical strength. These values are comparable to native cartilage tissue [30], making this composite scaffold particularly promising for cartilage tissue engineering applications.

Importantly, the enhanced mechanical performance of the CS40/SF/AV scaffold corresponds with the XRD results, which indicated increased semi-crystallinity with higher chitosan content. The XRD patterns showed broader but more pronounced peaks at 2θ = 20–25°, suggesting a more organized polymer structure in the scaffold matrix. This semi-crystalline organization may contribute to improved mechanical integrity, supporting the idea that scaffold microstructure, influenced by the CS content, plays a crucial role in determining compressive strength.

However, the significant reduction in mechanical properties under wet conditions (approximately one order of magnitude lower) highlights an important consideration for in vivo applications. Yan et al. [31] provided supporting evidence for this phenomenon. In their study of genipin-cross-linked collagen/chitosan scaffolds, they observed a tenfold decrease in mechanical strength when transitioning from dry to wet environments. This finding offers crucial insight into the challenges these biomaterials face under physiological conditions. Despite this reduction, CS40/SF/AV maintained relatively superior performance even in wet conditions, suggesting its potential suitability for cartilage environments.

**Figure 6:**
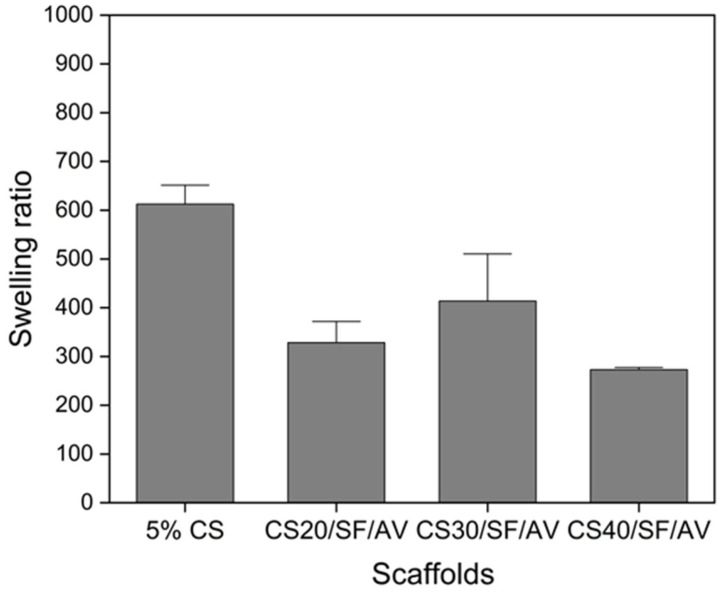
The swelling behavior of the scaffolds in PBS (at pH = 7.4, 37 °C).

Based on Figure 6, the swelling behavior of the scaffolds was analyzed in PBS (pH 7.4, 37 °C), revealing significant differences among various compositions. The control scaffold (5% CS) exhibited the highest swelling ratio of approximately 600%, while a notable reduction in swelling behavior was observed across all composite scaffolds upon incorporation of SF/AV components. The CS20/SF/AV scaffold, containing the highest concentration of SF/AV, demonstrated the most pronounced decrease in swelling capacity, with a ratio of 320%. The CS30/SF/AV scaffold showed an intermediate swelling ratio of 410%, while CS40/SF/AV exhibited the lowest swelling ratio of 270%.

The swelling behavior analysis provided crucial insights into the scaffold’s fluid uptake capacity and dimensional stability—factors that significantly impact cellular microenvironment, nutrient transport, and overall performance in cartilage tissue engineering [32]. While the control scaffold (5% CS) exhibited an excessive swelling ratio of approximately 600%, the incorporation of SF/AV components effectively modulated this behavior. Among the composite formulations, CS40/SF/AV demonstrated the most favorable swelling profile with the lowest ratio of 270%, while CS20/SF/AV showed an intermediate value (320%) and CS30/SF/AV exhibited a higher swelling ratio (410%). The CS40/SF/AV formulation’s moderate swelling capacity represents an optimal balance for cartilage tissue engineering applications. Excessive swelling, as observed in the control scaffold, can lead to a loss of structural integrity, reduced mechanical strength, and premature degradation [33]. Conversely, insufficient swelling may restrict nutrient diffusion and cellular activities. The CS40/SF/AV scaffold provides adequate hydration to facilitate cellular processes and nutrient exchange while maintaining dimensional stability and structural integrity necessary for load-bearing cartilage tissue. This balanced swelling behavior can be attributed to the higher proportion of chitosan, which forms stronger intermolecular interactions with SF/AV components, effectively restricting water molecule penetration between polymer chains while still accommodating essential fluid exchange. Furthermore, the controlled swelling of CS40/SF/AV scaffolds more closely mimics the hydration environment of native cartilage tissue, which typically maintains moderate fluid content while resisting excessive swelling under physiological conditions.

**Figure 7:**
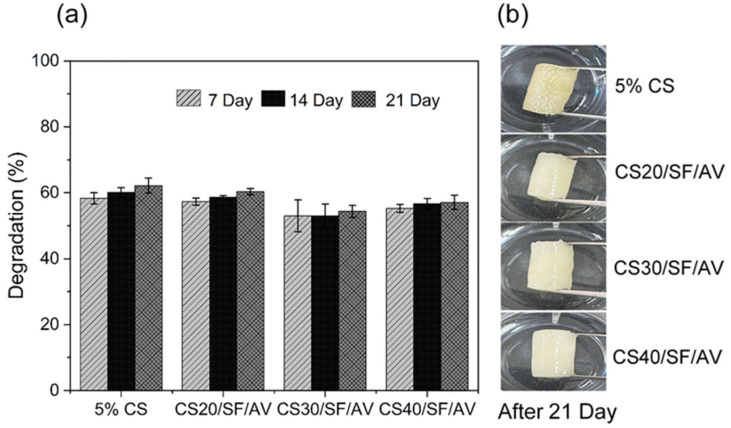
Biodegradation study of chitosan (CS) composite scaffolds in PBS (pH 7.4, 37 °C) over 21 days: (**a**) percentage degradation at 7, 14, and 21 days and (**b**) physical appearance of scaffolds after 21 days of immersion.

Figure 7 illustrates the biodegradation behavior of different scaffold compositions (5% CS, CS20/SF/AV, CS30/SF/AV, and CS40/SF/AV) in PBS containing lysozyme (pH = 7.4, 37 °C) over a 21-day period. All scaffold formulations exhibited a controlled degradation profile suitable for cartilage tissue engineering, with the majority of degradation occurring within the first two weeks and stabilizing thereafter. Among them, the CS40/SF/AV scaffold, which contained the highest chitosan content, demonstrated a balanced degradation profile with approximately 55% degradation at day 7, 56% at day 14, and 57% by day 21. This gradual degradation rate is favorable for supporting tissue regeneration, allowing sufficient time for new tissue formation while maintaining structural integrity [34,35].

These findings are consistent with the study by Bhardwaj et al. [36], who reported that incorporating silk fibroin into chitosan-based scaffolds reduced the degradation rate due to improved structural stability in lysozyme-rich environments. Gu et al. [37] demonstrated that composite scaffolds made from silk fibroin, gelatin, and chitosan possess favorable degradation characteristics and mechanical integrity under physiological conditions. These properties support chondrocyte proliferation and phenotype maintenance, confirming the scaffolds’ potential for cartilage regeneration. These comparative results underscore the importance of scaffold composition in tuning biodegradability to match the pace of tissue regeneration in cartilage engineering applications.

The biodegradation mechanism of the scaffolds primarily involves enzymatic hydrolysis of chitosan by lysozyme, which cleaves the β (1→4) glycosidic linkages between N-acetyl-D-glucosamine and D-glucosamine units in the chitosan backbone [38,39]. This enzymatic activity leads to chain scission, resulting in a reduction in molecular weight and eventual mass loss. The degree of deacetylation and crystallinity of chitosan significantly influence its susceptibility to enzymatic attack, with higher deacetylation enhancing degradation due to increased accessibility to lysozyme [40]. Moreover, the incorporation of silk fibroin enhances structural integrity via β-sheet formation, which confers resistance to enzymatic hydrolysis [41]. *Aloe vera*, rich in polysaccharides and bioactive compounds, may further stabilize the scaffold through hydrogen bonding and interfacial interactions, thereby modulating the degradation rate [16]. These compositional effects collectively govern the degradation behavior, enabling gradual scaffold resorption that aligns with the pace of new tissue formation.

Collectively, these findings demonstrate that the properties of CS/SF/AV composite scaffolds can be tailored by adjusting the relative proportions of the components. Among the tested formulations, CS40/SF/AV exhibited an optimal combination of porosity, mechanical strength, swelling behavior, and degradation rate, making it particularly suitable for cartilage tissue engineering. Additionally, Table 2 summarizes other studies on the mechanical properties of composition scaffolds. The enhanced mechanical properties observed in this formulation are attributed to the higher chitosan content, while the addition of silk fibroin and *Aloe vera* contributes bioactive functionality and structural stability.

To further verify the biocompatibility and cellular performance of CS40/SF/AV, in vitro studies were conducted including MTT assays, scanning electron microscopy (SEM) for cell morphology, and DAPI-stained fluorescence imaging. These evaluations confirmed the scaffold’s ability to support cell attachment, proliferation, and the maintenance of the chondrocyte phenotype, all of which are essential for successful cartilage regeneration. The consistent selection of CS40/SF/AV for detailed cellular analysis underscores its promising potential as a candidate for further in vivo studies and clinical translation.

### 3.3. Effects of Hydrogel on Chondrocyte Characteristic

**Figure 8:**
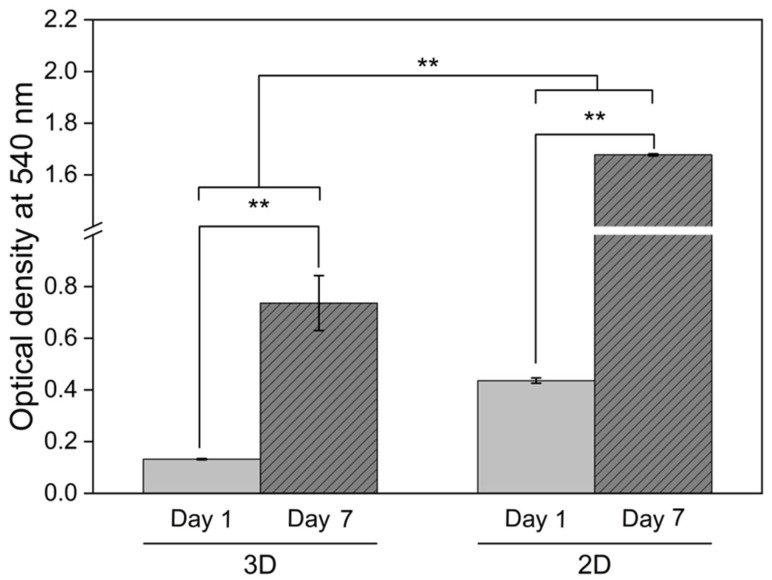
Chondrocyte viability assessment via MTT assay comparing conventional monolayer culture (2D) and CS40/SF/AV scaffold encapsulation (3D) at days 1 and 7. Measurements show formazan absorbance at 540 nm, with bars representing mean ± S.D. from triplicate experiments (*n* = 3). Statistical analysis revealed significant differences between day 1 and day 7 within each culture system (** *p* < 0.01, independent *t*-test). Additionally, two-way ANOVA indicated significant differences between the 2D and 3D culture environments (** *p* < 0.01).

The viability and proliferation of chondrocytes cultured under different conditions were assessed using the MTT assay, with optical density values at 540 nm measured on days 1 and 7 of cultivation (Figure 8). Both 2D monolayer and 3D CS40/SF/AV scaffold cultures demonstrated significant increases in optical density over time (*p* < 0.01), indicating active cell proliferation. However, two-way ANOVA revealed that cells cultured in the 2D monolayer exhibited significantly higher optical density than those in 3D scaffolds at day 7 (*p* < 0.01), suggesting different proliferation characteristics between the two culture systems.

This trend aligns with the findings of Ngoenkam et al. [44], who observed that chondrocytes in 2D culture gradually adopted an elongated, fibroblastic morphology, as shown by light microscopy and SEM. Such morphological transition is associated with dedifferentiation and promotes rapid cell expansion. Our results support this observation, suggesting that the increased proliferation in 2D may be linked to altered cell shape and cytoskeletal organization, which favor mitosis.

In contrast, chondrocytes within the 3D CS40/SF/AV scaffolds maintained a rounded, spherical morphology, characteristic of native cartilage cells. According to Buckwalter and Mankin [45], while this morphology is essential for maintaining the chondrogenic phenotype, it inherently limits cell proliferation due to spatial and cytoskeletal constraints. Thus, although the 3D scaffold provides a more biomimetic environment conducive to cartilage-specific function, it supports a slower rate of proliferation compared to 2D culture.

These findings highlight a fundamental trade-off in scaffold design between preserving the cellular phenotype and supporting controlled cell proliferation, an important consideration in the development of effective cartilage tissue engineering strategies.

**Figure 9:**
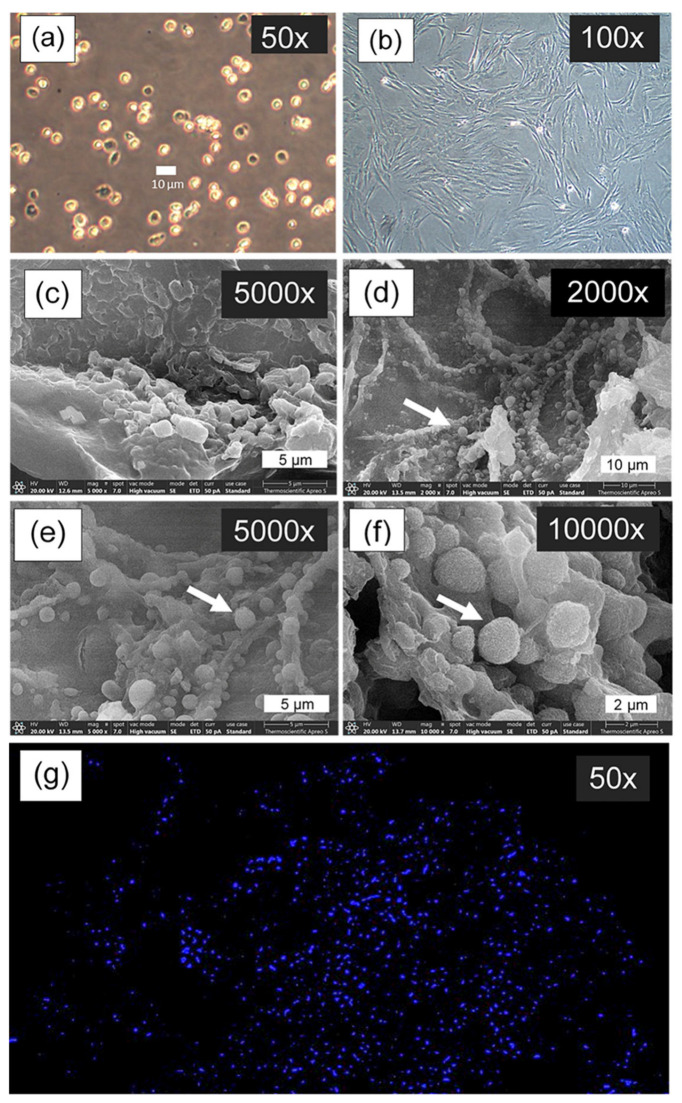
Cell morphology imaged by inverted microscopy: cell suspension culture (**a**), and chondrocytes cultured on the tissue culture plate (2D culture) for 7 days (**b**); SEM micrograph of CS40/SF/AV scaffold without seeding cell culture (**c**); SEM micrograph of chondrocyte cultured on the CS40/SF/AV scaffold for 7 days (3D culture, (**d**–**f**)), where white arrows indicate individual chondrocyte cells attached to and integrated with scaffold structure; and fluorescence microscopy image of chondrocyte nuclei stained with DAPI after 28 days of culture on CS40/SF/AV scaffold (**g**).

The chondrocyte morphology analysis revealed distinctive cellular characteristics across different culture conditions and time points, as shown in Figure 9. Cell suspension culture (Figure 9a) observed under inverted microscopy at 50× magnification showed spherical chondrocytes with typical rounded morphology. In 2D culture (Figure 9b), chondrocytes observed under inverted microscopy at 100× magnification after 7 days of culture on tissue culture plates demonstrated characteristic fibroblast-like morphology, with elongated appearance. Figure 9c showed a SEM micrograph of the CS40/SF/AV scaffold without cell seeding at 5000× magnification for comparison. For 3D culture on the CS40/SF/AV scaffold, SEM micrographs at different magnifications (2000×, 5000×, and 10,000×) demonstrated the progressive development of chondrocyte morphology after 7 days (Figure 9d–f). The cells initially appeared scattered on the scaffold surface and exhibited spherical and spindle-shaped morphology with filopodial extensions [46]. The white arrows in the SEM micrographs indicate the presence of individual chondrocytes adhering to the scaffold structure. At higher magnification (10,000× in Figure 9f), the detailed surface topography revealed that the chondrocytes maintained their characteristic spherical morphology while interacting with the scaffold matrix. The chondrocytes exhibited successful attachment and integration with the scaffold surface, indicating good cell-material compatibility, as also reported in the study by Chicatun et al. [47], which demonstrated the material’s suitability for chondrocyte culture. These cultured chondrocytes displayed typical morphological dimensions that align with the expected parameters for articular cartilage cells. This morphological assessment confirms that the CS40/SF/AV scaffold provides a suitable three-dimensional environment for chondrocyte attachment and maintenance of their characteristic cellular morphology, which is crucial for their biological function and ECM production.

Figure 9g presents DAPI (4′,6-diamidino-2-phenylindole) staining, a fluorescent dye that binds specifically to DNA in cell nuclei, visualized as bright blue dots against a dark background. The image illustrates the distribution of chondrocytes on the CS40/SF/AV scaffold, where the nuclei are seen uniformly scattered throughout the three-dimensional matrix. This pattern indicates successful cell seeding, attachment, and proliferation. Consistent with the findings of Huang et al. [48] and Rikkers et al. [49], the uniform nuclear distribution suggests that the scaffold provides a favorable environment for chondrocyte viability and growth.

## 4. Conclusions

This study successfully developed composite hydrogel scaffolds composed of chitosan, silk fibroin, and *Aloe vera* gel extract for cartilage tissue engineering. Among the formulations tested, CS40/SF/AV (3.00% chitosan, 2.70% silk fibroin, 0.075% *Aloe vera*) exhibited the most favorable properties, including high porosity (72.23%), optimal pore diameter (79.57 μm), and enhanced mechanical strength. It also showed balanced swelling behavior (270%) and a controlled degradation rate (57% over 21 days), indicating good structural stability for regenerative applications. Characterization by FTIR and XRD confirmed successful component integration and a semi-crystalline structure. In vitro experiments demonstrated that CS40/SF/AV scaffolds supported chondrocyte attachment and viability while preserving their spherical morphology. The synergistic combination of these natural biomaterials provided a biocompatible microenvironment with a well-balanced profile of mechanical support and bioactivity. Overall, the CS40/SF/AV scaffold shows strong potential as a biomaterial platform for cartilage tissue engineering. Future studies should focus on long-term culture performance and in vivo evaluation.

## 5. Patents

The petty patent entitled “A process for producing hydrogel scaffold materials composed of chitosan, fibroin and *Aloe vera* gel extract for cartilage cell adhesion and function” is undergoing a review process. Date received: 1 November 2024. Application Number: 2403003662.

## Figures and Tables

**Table 1:** Preparation of scaffolds with different concentrations. Chitosan, silk fibroin, and *Aloe gel* extract were mixed in different ratios to form hydrogel. The five percent of chitosan served as control group by adding water, without natural extracts.

Sample Code Used in This Study	CS:SF:AV	CS (% *w*/*v*)	SF (% *w*/*v*)	AV (% *w*/*v*)	Cross-Linker (% *w*/*v*)
5% CS	-	5	-	-	2.25
CS20/SF/AV	20:55.46:1.54	1.50	4.16	0.12	2.25
CS30/SF/AV	30:45.73:1.27	2.25	3.43	0.10	2.25
CS40/SF/AV	40:36:1	3.00	2.70	0.075	2.25

**Table 2:** Studies on the mechanical properties of composition scaffolds.

Composition	Porosity (%)	Pore Size (μm)	Compressive Strength (Dry) (MPa)	Compressive Modulus (Dry) (MPa)	Swelling Ratio (%)	Degradation Rate (%)	Ref.
3.00% wt CS, 2.70% wt SF, 0.075% wt AV	72.23 ± 4.85%	79.57 ± 3.68	0.75 ± 0.15	6.67 ± 1.44	270	55–57% over 21 days	This study
50%wt Chitosan, 50%wt Agarose	93	150–300	0.35 ± 0.03	4.5 ± 0.4	approximately 1500	not reported (stable in PBS)	[17]
Chitosan, Gelatin, 10%wt nHA, 2%wt PANI	83.6	approximately 200	5.06	25.45	approximately 1200	approximately 45 (over 21 days)	[19]
16%wt Silk Fibroin, 10%wt HAP	86.2 ± 1.59	approximately 200	0.3	not reported	5.23 ± 0.06	not reported	[42]
3:7 Ratio CS:Gel with 0.7 Fraction ꞵ-TCP	approximately 92–98	323 ± 104	0.88 ± 0.05	10.9 ± 3.5	not reported	gradual degradation over 12 weeks in vivo	[43]

## Data Availability

Data are contained within the article.
